# Endovascular Treatment of Coarctation of the Aorta with a Self-Expanding Endoprosthesis: How I Do It Using the Braile Dominus^®^ Coarctation Aorta Device

**DOI:** 10.21470/1678-9741-2020-0345

**Published:** 2021

**Authors:** Rodrigo Petersen Saadi, Eduardo Keller Saadi, Ana Paula Tagliari, Marina Petersen Saadi

**Affiliations:** 1 Department of Cardiovascular Surgery, Hospital de Clínicas de Porto Alegre, Porto Alegre, Rio Grande do Sul, Brazil.; 2 Postgraduate Program in Cardiology and Cardiovascular Sciences, Universidade Federal do Rio Grande do Sul (UFRGS), Porto Alegre, Rio Grande do Sul, Brazil.; 3 Department of Cardiovascular Surgery, Hospital São Lucas da Pontifícia Universidade Católica do Rio Grande do Sul (PUCRS), Porto Alegre, Rio Grande do Sul, Brazil.; 4 Department of Cardiology, Hospital de Clínicas de Porto Alegre, Porto Alegre, Rio Grande do Sul, Brazil.

**Keywords:** Aortic Coarctation, Computed Tomography Angiography, Aortic Rupture, Coarctation, Endovascular Procedures, Prosthesis and Implants

## Abstract

The coarctation of the aorta is a relatively highly prevalent congenital heart disease and may be diagnosed as an underline cause of hypertension in adolescents and adults. The gold standard treatment for coarctation of the aorta in these patients is being replaced - from open surgery to endovascular therapy. Some prostheses have been developed to treat the coarctation with less acute and chronic complications. The Dominus^®^ Coarctation Aorta (Braile Biomédica) is the first self-expandable prosthesis created specifically to treat coarctation of the aorta, reducing possible acute complications, like aortic rupture or aortic dissection. Here, we discuss the step-by-step method for using this prosthesis.

**Table:** 

Abbreviations, acronyms & symbols	
BA	= Balloon angioplasty
CHD	= Congenital heart disease
CoA	= Coarctation of the aorta
CTA	= Computed tomography angiography
PPG	= Peak pressure gradient
US	= Ultrasound

## INTRODUCTION

With an incidence of 0.2-0.6 in 1,000 live births, coarctation of the aorta (CoA) is the sixth most prevalent congenital heart disease (CHD), corresponding to 5-8% of all CHD ^[1,2]^. Depending on the balance between flow disturbance and compensatory mechanisms, CoA clinical presentation may vary from a critically ill neonate to an asymptomatic child or a hypertensive adult ^[3,4]^. Despite this variable clinical spectrum, CoA invariably results in increased left ventricle afterload, upper body hypertension, and lower body decreased perfusion and pressure.

Trying to modify CoA poor prognosis, in 1944, Crafoord performed the first CoA surgical intervention. Since then, new endovascular alternatives have arrived with the premise of bringing a similarly effective but less invasive approach to manage larger children and adults with CoA.

Herein, we discuss, in a step-by-step manner, CoA endovascular treatment in adolescents and adults using the Dominus® Coarctation Aorta endoprosthesis (Braile Biomédica, São José do Rio Preto, SP).

### Dominus® Coarctation Aorta Endoprosthesis

**Fig. 1:**
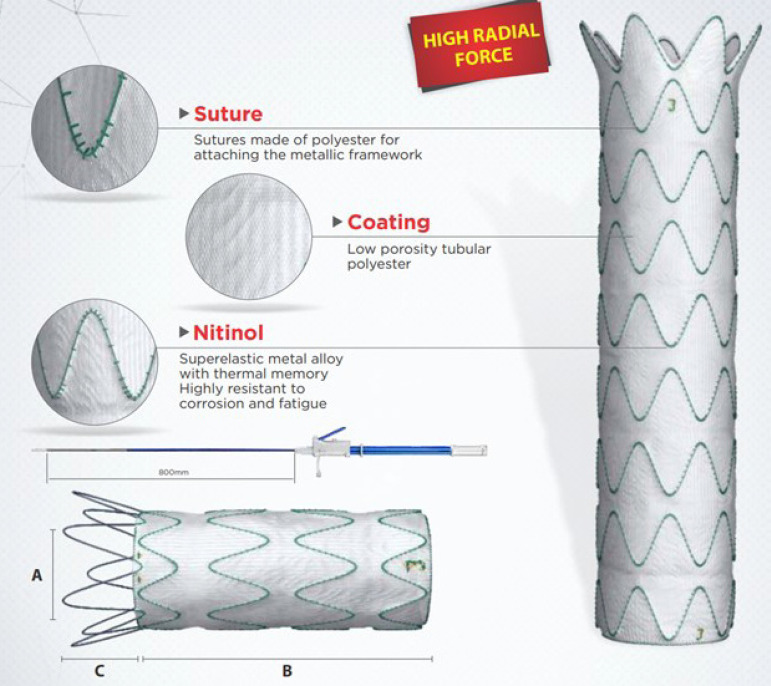
Braile Dominus® Coarctation Aorta endoprosthesis characteristics.

The Dominus® device consists of a self-expanding metallic stent-graft, coated with a low-porosity polyester and nitinol structure. It is particularly indicated for CoA endovascular correction in adolescents and adults. The stent-graft may present a non-covered proximal stent module, allowing better prosthesis fixation, known as "Free-Flow"; or it may be composed of a module partially covered with tissue following the shape of the metal structure, called "Open-Web" (Figure 1). Regarding Dominus®' fixation mechanism, the nitinol superelastic metal alloy with thermal memory provides a high radial force and resistance to corrosion and fatigue. It is available in 18-, 20-, and 22-mm diameter × 50- or 70-mm length, specially designed to treat CoA, compatible with an 18Fr sheath.

## TECHNIQUE

### Preprocedural Planning (Figure 2A)

Computed tomography angiography (CTA) of the thoracic aorta, abdominal aorta, and iliac vessels is essential to allow proper procedural planning and safe intraprocedural device delivery. Some relevant measurements are the aorta diameter proximally and distally to the CoA zone, CoA extension, minimal aorta diameter at the CoA zone, as well as iliac and femoral vessels access, which should have a minimum diameter of 5.5 mm.

**Fig. 2:**
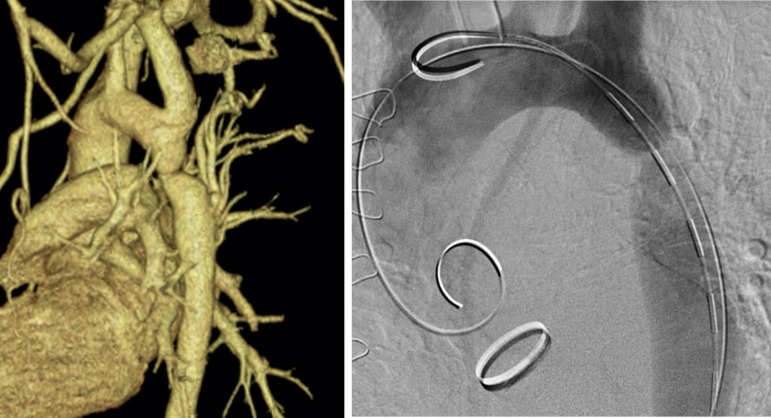
A) Preprocedural computed tomography angiography revealing coarctation of the aorta just at the origin of the left subclavian artery; B) Intraprocedural angiography confirming the previous findings.

As the endoprosthesis anchors in the narrower aorta zone, no oversizing regarding the native aorta diameter is usually recommended.

The choice of the stent type depends on some anatomical factors, such as proximity of native coarctation to head and neck vessels, presence of a concomitant aneurysm, patient size, operator preference, and device availability.

### Procedural Steps

Under general anesthesia, accesses to both common femoral arteries are established by open dissection or puncture guided by ultrasound (US), fluoroscopy, or anatomical landmarks. The femoral artery with the largest caliber, less tortuosity, and less calcification should be chosen as the primary device navigation side. In contrast, the contralateral side is used for pigtail insertion. In patients with challenging femoral access, an alternative radial artery puncture could be used for the pigtail insertion. After obtaining the arterial accesses, intravenous heparin is administered at 1 mg/Kg to achieve an activated clotting time > 250 seconds.

Through the primary femoral access, after applying the preclosure technique using a Perclose/ProGlide device (Abbott Vascular, California, United States of America), a 5Fr straight-tip catheter (vertebral or multipurpose) is inserted and advanced into the descending aorta over a soft-tipped hydrophilic 0.035" guidewire. During the CoA zone crossing, extreme caution should be taken to avoid any aortic injury. In the case of a challenging crossing, 0.014" hydrophilic guidewire can be used as an alternative to the 0.035". If the CoA zone has a diameter < 4-5 mm or the 5Fr catheter cannot cross it, a small balloon (6 × 20 mm) can be used to perform a pre-dilatation.

After this step, the catheter is advanced into the ascending aorta, close to the aortic valve. The hydrophilic guidewire is exchanged by an extra or super stiff wire (for example, an Amplatz extra or super stiff or a Lunderquist extra stiff Double Curved).

The calibrated pigtail catheter is then positioned in the aortic arch, just proximal to the lesion, and used to perform a control angiography (Figure 2B). This initial angiography is usually obtained with 30 ml of contrast, 15 ml/second with 800 PSI pressure, in a left anterior oblique fluoroscopy projection (45 to 70 degrees), and slight cranial fluoroscopy projection. The aim of this control angiography is to visualize the aortic arch, supra-aortic vessels, mainly the left subclavian artery, aortic isthmus, CoA zone, and distal aorta. Rarely, a balloon angioplasty (BA) is performed alone, which is usually reserved for small children ^[4]^.

**Fig. 3:**
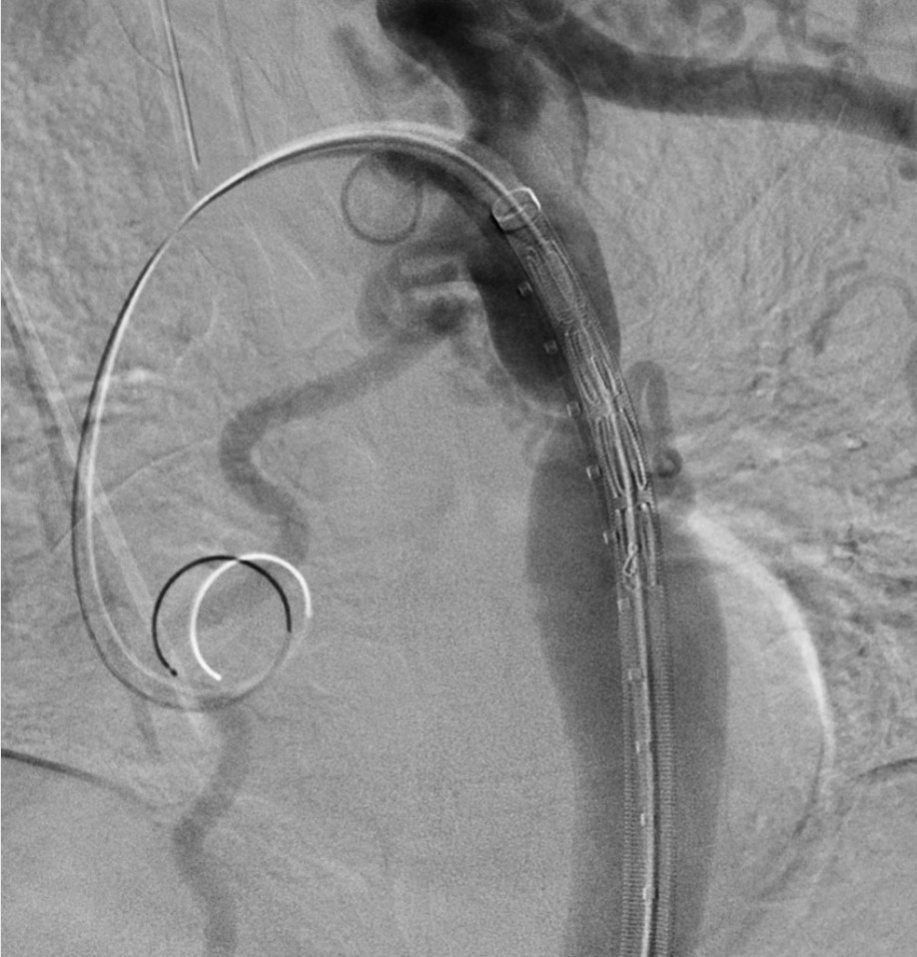
Dominus® Coarctation Aorta delivery system advanced retrogradely until the tip of the sheath is slightly beyond the coarctation zone.

**Fig. 4:**
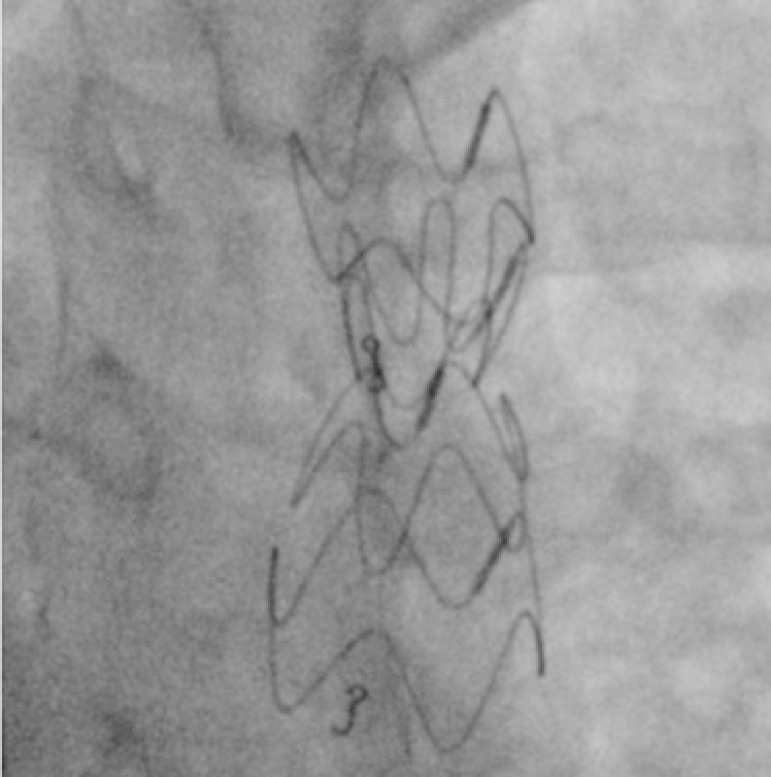
Dominus® Coarctation Aorta endoprosthesis released under fluoroscopic control, with no need of post-dilatation. Magnification shows the expanded prosthesis.

In order to implant the endoprosthesis, the small sheath is exchanged for the selected long delivery sheath, which is advanced retrogradely until the tip of the sheath is slightly beyond the lesion zone (Figure 3). The endoprosthesis is positioned just after the left subclavian artery origin, and a control angiography confirms its proper position. In the next step, the sheath is retracted and the Dominus® Coarctation Aorta endoprosthesis is released under strict fluoroscopic control (Figure 4). A final angiography is performed to assess the result and to exclude any complications. Finally, the delivery system is removed, and the vascular access is repaired by a percutaneous (Perclose/ProGlide) or open suture, or manual compression (not recommended).

**Fig. 5:**
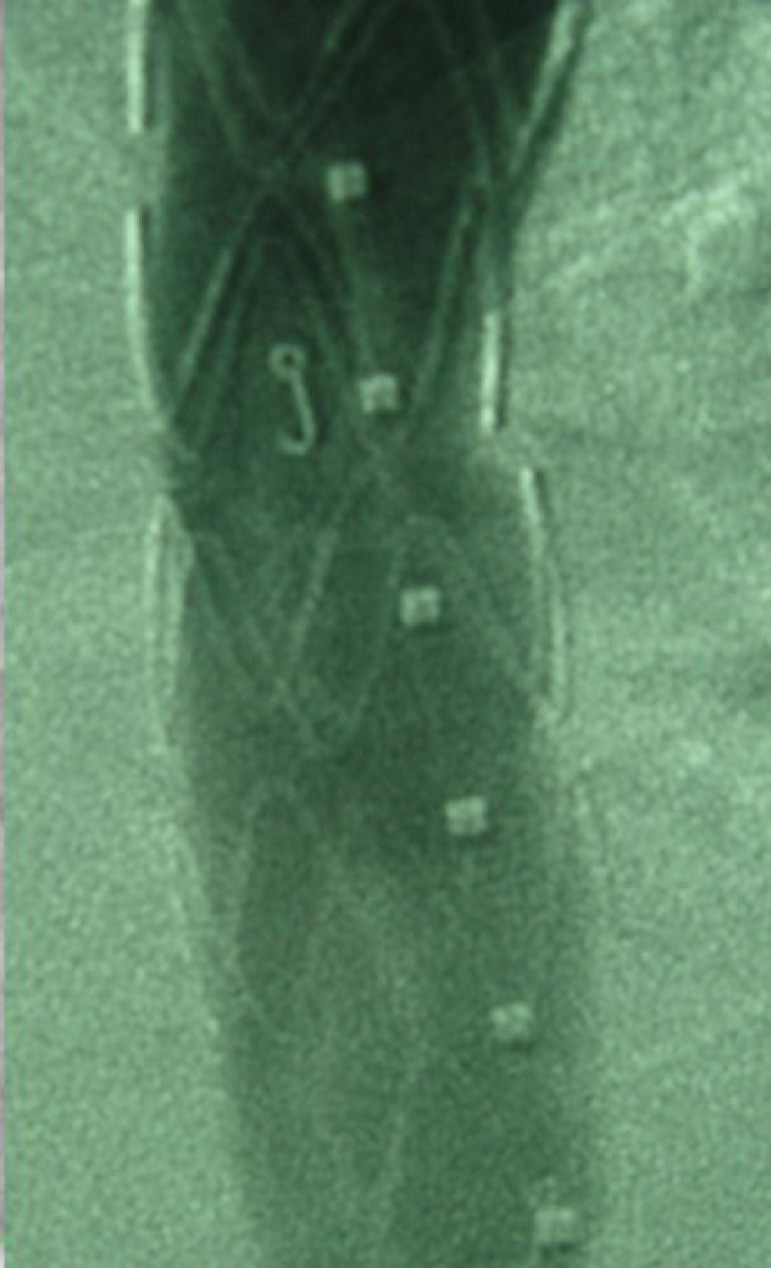
Control aortography performed six months after the procedure showing the full expansion of the Dominus® Coarctation Aorta endoprosthesis.

Successful device implantation is defined as a reduction in the gradient to < 20 mmHg or < 10 mmHg, varying according to different literature sources, or a ratio of post-stent coarctation to descending aorta gradient > 0.8 ^[5]^. Post-dilatation can be performed if CoA relief or stent apposition to the aortic wall is deemed suboptimal. As a general rule, post-dilatation is not recommended if the systolic gradient is < 10 mmHg. This proposition is based on the fact that, just after a few weeks or months, the self-expanding prosthesis will achieve its full expansion and, therefore, its final maximal diameter (Figure 5). This can be confirmed with control CTA, performed during the follow-up.

After a successful procedure, the patients can be discharged in one or two days, receiving antiplatelet therapy (aspirin 100 mg daily), and oriented to keep a rigid blood pressure control. Chest radiograph, electrocardiogram, and transthoracic echocardiogram are obtained before hospital discharge.

## DISCUSSION

According to the most recent American and European Guidelines for the Management of Adults with CHD, surgical or transcatheter CoA interventions are indicated in all patients with a peak pressure gradient (PPG) > 20 mmHg, regardless of symptoms but with upper limb hypertension (> 140/90 mmHg in adults), pathological blood pressure response during exercise, or significant left ventricle hypertrophy (class I, level C). Independent of the PPG, hypertensive patients with ≥ 50% aortic narrowing relative to the aortic diameter at the diaphragm level should also be considered for treatment (class IIa, level C) ^[6,7]^.

Native CoA has historically been treated by surgery. Recently, however, transcatheter approaches have been increasingly refined, in part because of increased operator experience, and also because of improved balloon and stent technology, which have improved the safety and success of CoA transcatheter approach ^[5]^.

Nowadays, a wide variety of stents are available, varying in flexibility degree, profile, foreshortening on expansion, maximal expansion diameter, and radial strength. Herein, we present a step-by-step method to guide the new Dominus® Coarctation Aorta implantation. Besides its specific design to manage CoA, this self-expanding covered stent is available in different models, with Free-Flow or Open-Web designs, expanding its range of treatment.

When compared to BA, stents are known to be more advantageous in improving luminal diameter, providing minimal residual PPG, and sustaining the hemodynamic results. Stents radial force also prevents vessel wall elastic recoil and may tack intimal flaps to the aortic wall, potentially allowing healing and reducing the risk of aortic dissection and aneurysm formation ^[5,8,9]^. In the setting of risk of aortic wall injury, covered stents are particularly useful as it was demonstrated by the Coarctation of the Aorta Stent Trial II. In this study, among the 158 patients treated with covered stents, a decrease in PPG from 27 mmHg to 4 mmHg, aligned to an overall success rate of 92%, and no aortic wall injury, need for reintervention, or death was reported. Despite this excellent efficacy, four patients presented important access site vascular injury, which made the authors point out that the larger sheath required to deliver the covered stent may increase vascular complications ^[10]^.

When using the Dominus® endoprosthesis, we have some concerns about peripheral access injury risk, since, despite its low risk of immediate complications, the 18Fr delivery system can increase the vascular damage risk. Considering this fact, we recommend extreme caution in obtaining arterial accesses and proper training in suture-mediated vascular closure devices, if this technology is used. Moreover, US-guided puncture can be used as an adjuvant approach in patients with no palpable pulses.

Regardless of this concern, we believe that the Braile Dominus® Coarctation Aorta endoprosthesis presents some advantages such as a lower risk of aortic rupture or dissection because it is not necessary to achieve an immediate perfect anatomic result. As a consequence of the continuous and slow expansion of the Nitinol frame, its maximum diameter will be achieved in some weeks or months after the procedure. Furthermore, it has a short learning curve, an affordable price, and, hence, the potential to achieve a spread use in CoA transcatheter management.

## CONCLUSION

Careful preprocedural planning, proper operator training, consolidated experience in endovascular procedures, and adequate patient and prosthesis selection are the main steps for a successful transcatheter CoA treatment. Self-expandable endograft is a useful alternative to covered stents to treat adolescents and adults with CoA.

**Table:** 

Authors' roles & responsibilities	
RPS	Substantial contributions to the conception or design of the work; or the acquisition, analysis, or interpretation of data for the work; drafting the work or revising it critically for important intellectual content; agreement to be accountable for all aspects of the work in ensuring that questions related to the accuracy or integrity of any part of the work are appropriately investigated and resolved; final approval of the version to be published
EKS	Substantial contributions to the conception or design of the work; or the acquisition, analysis, or interpretation of data for the work; drafting the work or revising it critically for important intellectual content; agreement to be accountable for all aspects of the work in ensuring that questions related to the accuracy or integrity of any part of the work are appropriately investigated and resolved; final approval of the version to be published
APT	Substantial contributions to the conception or design of the work; or the acquisition, analysis, or interpretation of data for the work; drafting the work or revising it critically for important intellectual content; agreement to be accountable for all aspects of the work in ensuring that questions related to the accuracy or integrity of any part of the work are appropriately investigated and resolved; final approval of the version to be published
MPS	Substantial contributions to the conception or design of the work; or the acquisition, analysis, or interpretation of data for the work; drafting the work or revising it critically for important intellectual content; agreement to be accountable for all aspects of the work in ensuring that questions related to the accuracy or integrity of any part of the work are appropriately investigated and resolved; final approval of the version to be published
